# Superior ability of dietary fiber utilization in obese breed pigs linked to gut microbial hydrogenotrophy

**DOI:** 10.1093/ismeco/ycaf043

**Published:** 2025-03-09

**Authors:** Xuan Li, Chunlong Mu, Haiqin Wu, Erwin G Zoetendal, Ruihua Huang, Kaifan Yu, Weiyun Zhu

**Affiliations:** Laboratory of Gastrointestinal Microbiology, Jiangsu Key Laboratory of Gastrointestinal Nutrition and Animal Health, College of Animal Science & Technology, Nanjing Agricultural University, Nanjing, Jiangsu 210095, China; National Center for International Research on Animal Gut Nutrition, Nanjing Agricultural University, Nanjing, Jiangsu 210095, China; Department of Biochemistry and Molecular Biology, Cumming School of Medicine, University of Calgary, Calgary, Alberta T2N 1N4, Canada; Laboratory of Gastrointestinal Microbiology, Jiangsu Key Laboratory of Gastrointestinal Nutrition and Animal Health, College of Animal Science & Technology, Nanjing Agricultural University, Nanjing, Jiangsu 210095, China; National Center for International Research on Animal Gut Nutrition, Nanjing Agricultural University, Nanjing, Jiangsu 210095, China; Laboratory of Gastrointestinal Microbiology, Jiangsu Key Laboratory of Gastrointestinal Nutrition and Animal Health, College of Animal Science & Technology, Nanjing Agricultural University, Nanjing, Jiangsu 210095, China; National Center for International Research on Animal Gut Nutrition, Nanjing Agricultural University, Nanjing, Jiangsu 210095, China; Laboratory of Microbiology, Wageningen University & Research, Wageningen 6700 HB, The Netherlands; Laboratory of Gastrointestinal Microbiology, Jiangsu Key Laboratory of Gastrointestinal Nutrition and Animal Health, College of Animal Science & Technology, Nanjing Agricultural University, Nanjing, Jiangsu 210095, China; National Center for International Research on Animal Gut Nutrition, Nanjing Agricultural University, Nanjing, Jiangsu 210095, China; Laboratory of Gastrointestinal Microbiology, Jiangsu Key Laboratory of Gastrointestinal Nutrition and Animal Health, College of Animal Science & Technology, Nanjing Agricultural University, Nanjing, Jiangsu 210095, China; National Center for International Research on Animal Gut Nutrition, Nanjing Agricultural University, Nanjing, Jiangsu 210095, China; Laboratory of Gastrointestinal Microbiology, Jiangsu Key Laboratory of Gastrointestinal Nutrition and Animal Health, College of Animal Science & Technology, Nanjing Agricultural University, Nanjing, Jiangsu 210095, China; National Center for International Research on Animal Gut Nutrition, Nanjing Agricultural University, Nanjing, Jiangsu 210095, China

**Keywords:** gut microbiome, obesity, hydrogenotrophic microbes, methanogenesis, fiber utilization

## Abstract

Dietary fiber is widely recognized for its benefits to human health. Individual variations in the ability to degrade dietary fiber are influenced by the gut microbiome that may be associated with the host’s metabolic phenotype and genetic diversity. This is exemplified by the distinct fiber digestibility observed in obese (e.g. Meishan) and lean-breed (e.g. Yorkshire) pigs. However, the underlying mechanisms remain unclear. The present study found that with the same diet under the same environment, the obese-type Meishan pigs showed greater dietary fiber digestibility and harbored higher abundances of polysaccharide-degrading bacteria (*Bacteroides*, *Treponema*, and *Paraprevotella*) compared to lean-type Yorkshire pigs. Metatranscriptomic profiling revealed that the elevated presence of *Bacteroides* contributed to the enrichment of carbohydrate-active enzymes, particularly those degrading arabinoxylan, indicating a preference for arabinoxylan as a substrate in Meishan pigs. Further enzymatic-product measurements, combined with microbial enzyme profiles, validated greater microbial conversion of xylose into short-chain fatty acids (SCFAs) in Meishan pigs. Additionally, higher abundances of hydrogenotrophic microbes (*Methanobrevibacter* and *Blautia*) were detected in the Meishan gut, along with the enrichment of methanogenesis and acetogenesis pathways. To determine whether methanogenesis drives inter-breed variation in arabinoxylan degradation, an *in vitro* experiment using the methanogen inhibitor, 2-bromoethanesulfonate (BES) was performed. The results confirmed that Meishan gut microbiome effectively reduced hydrogen accumulation through methanogenesis, promoting arabinoxylan degradation. Conversely, inhibiting methanogenesis by BES led to hydrogen accumulation, reduced SCFAs, β-xylosidase activity, and *Bacteroides* abundances. These findings demonstrate that the Meishan pigs have a superior ability of dietary fiber utilization with greater microbial conversion to more SCFAs, which is linked to stronger hydrogenotrophic methanogenesis. This study reinforces the role of gut microbial hydrogenotrophy in dietary fiber utilization in pigs.

## Graphical Abstract

**Graphical Abstract:**
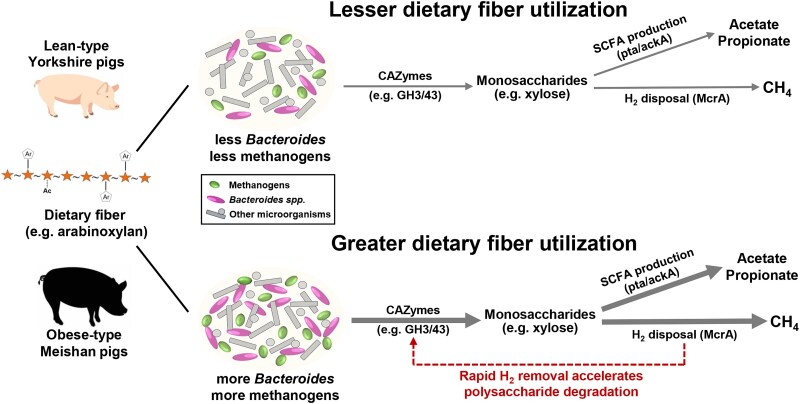

## Introduction

Dietary fiber contains a variety of structurally complex plant polysaccharides that are undigested by the host enzymes in the small intestine and reach the colon, where they can be utilized by resident microbes. The gut microbiome encodes a large repertoire of carbohydrate-active enzymes (CAZymes) responsible for the breakdown of indigestible plant polysaccharides into oligosaccharides or monosaccharides [1]. These sugars are then fermented primarily into short-chain fatty acids (SCFAs) and gasses such as CO_2_, H_2_, and CH_4_. SCFAs, including acetate, propionate, and butyrate, are estimated to contribute to 10% of the body’s energy requirements [2] and also serve as key signaling molecules affecting various physiological processes [3]. Accumulating evidence suggests that microbial-derived SCFAs mainly mediate the beneficial effects of dietary fiber [4, 5].

Degradation of plant polysaccharides involves multiple metabolic interactions among different microbial groups. One of the fundamental processes is hydrogen disposal during microbial cross-feeding. The hydrogen generated by polysaccharide-degrading bacteria is disposed by hydrogenotrophic microbes, including methanogens, acetogens, and sulfate-reducing bacteria (SRB) [6]. Efficient hydrogen disposal could reduce hydrogen partial pressure and maintain bacterial nicotinamide adenine dinucleotide dehydrogenases to support appropriate polysaccharide degradation [7, 8]. Studies based on *in vitro* co-cultures showed that hydrogen disposal by the methanogen in the human gut enhances the growth and metabolic efficiency of certain polysaccharide-degrading bacteria, such as *Bacteroides* and *Ruminococcus* species [9, 10]. A gnotobiotic mouse study has demonstrated that the presence of methanogen promotes bacterial degradation of dietary fructans and increases SCFA production [11]. As such, hydrogenotrophic methanogens have a critical impact on microbial composition and polysaccharide degradation.

Individual variations in dietary fiber degradation are common traits shared by humans and animals. The ability to ferment plant polysaccharides into SCFAs differs considerably among individuals receiving the same dietary fiber [12–15]. Animal studies have shown that intestinal fiber digestibility varies across pig breeds regardless of diets [16, 17]. Such variability in polysaccharide degradation may depend on the gut microbial differences that are related to the host’s genetic diversity [18] and metabolic phenotype [19]. It has been proposed that the gut microbiomes in obese and lean mice differ in composition and their ability to extract energy from the diet [20]. Further studies revealed that methanogens are one of the functional groups that vary between obese and lean phenotypes. For instance, a quantitative real-time polymerase chain reaction (qPCR) analysis detected higher numbers of gut methanogens in three obese individuals compared to three normal-weight individuals [21]. Interestingly, high levels of gut methanogens and exhaled methane are associated with higher body mass index in large cohorts of humans [22–24]. However, subsequent studies reported inconsistent or even converse findings with less or no relevance between methanogens and obesity [25, 26]. Thus, the role of methanogens in obesity has yet to be elucidated. While many studies focus on microbial composition including methanogens, SCFA concentration, and their association, the microbial conversion process from diet to the energy source SCFA, as well as the hydrogenotrophic pathways in obese and lean phenotypes, remains unclear.

Pigs share a high similarity with humans in their digestive physiology and allow easier manipulation of environmental exposure than humans [27]. Obese-breed Meishan pigs have a greater capacity to digest dietary fiber than lean-breed Yorkshire pigs [16]. Meishan and Yorkshire piglets also differ in their bacterial and methanogen species [28, 29], further raising interest in dissecting their link with polysaccharide degradation. Here, we employed genetically distinct Meishan and Yorkshire pigs with the same diet under the same environment, and by using 16S rRNA sequencing, metatranscriptomic, and targeted metabolomic approaches, we found that the gut microbiomes of these two breed pigs exhibited distinct arabinoxylan-degrading enzyme activities, along with differences in SCFA production and hydrogenotrophic methanogenesis and acetogenesis pathways. *In vitro* experiments further validated that the greater capacity for arabinoxylan degradation in Meishan pigs is linked with increased hydrogenotrophic methanogenesis. These findings reveal that methanogen-bacteria interactions drive superior dietary fiber utilization in Meishan pigs. Given the widely recognized role of fiber utilization in gut health, the present study further provides insights into the significance of methanogens in intestinal metabolism.

## Materials and methods

### Animal experiment and sample collection

All animal procedures were approved by the Institutional Animal Care and Use Committee of the Nanjing Agricultural University (no. SYXK 2018-0071) in compliance with the relevant guidelines and regulations. Given the difference in growth rate and mature body weights between the two porcine breeds, the Meishan and Yorkshire pigs at the same physiological stage were selected for this experiment. The different pig breeds are considered to be at the same physiological stage when their body weights reach the same percentage of their corresponding mature body weights [30, 31]. Thus, a total of 12 finishing barrows, six from each Meishan (235 days of age, average body weight 65.73 ± 3.62 kg) and Yorkshire (171 days of age, average body weight 83.40 ± 2.67 kg) at a physiological stage of ~35% of their mature body weights were employed and raised on an experimental farm in Jiangsu Province. All pigs were maintained under the same environmental conditions and fed a corn-soybean-wheat bran diet. Diet details are listed in Supplementary Table S1. Each barrow was housed in a single pen with free access to food and water. The experiment lasted for 28 days. All pigs were euthanized using electrical stunning and sacrificed by exsanguination at the end of the experiment. The average backfat thickness was determined as the mean of measurements of the first rib, last rib, and last lumbar vertebrae at the midline using a sliding caliper. The intramuscular fat content in the longissimus muscle was measured according to the procedures of the Association of Official Analytical Chemist (AOAC). The digestive tract was immediately dissected, and the colon was separated, the digesta from the middle section of the colon was collected and snap-frozen by liquid nitrogen, and then stored at −80°C for further analysis.

### *In vitro* batch fermentation experiment

*In vitro* batch incubations were conducted to compare inter-breed variability in microbial hydrogen disposal and explore the role of methanogens in bacterial arabinoxylan degradation. Fresh fecal samples were collected from four Yorkshire and four Meishan pigs at the end of the animal feeding experiment. Fecal samples were sealed in plastic bags pre-filled with CO_2_ using an AnaeroPack (Mitsubishi Gas Chemical Co., Inc., Tokyo, Japan) and immediately transferred to an anaerobic incubator in the laboratory. Subsequently, fecal samples were diluted in sterile phosphate-buffered saline at a ratio of 1:10 (w/v) and filtered through four layers of sterile gauze. Finally, these fecal slurries were sealed in serum bottles and stored at 37°C until the inoculation.

Basal medium was prepared according to a previously described method [32]. Briefly, every 1 l of basal medium comprised the following components: 0.6 g KCl, 0.6 g NaCl, 0.2 g CaCl_2_·2H_2_O, 0.5 g MgSO_4_·7H_2_O, 1.46 g KH_2_PO_4_, 3.55 g Na_2_HPO_4_, 1.0 g Trypticase, 0.54 g NH_4_Cl, 10 mL trace elements, 10 ml hemin, 1 ml resazurin, 50 ml NaHCO_3_, 1 ml vitamin/phosphate solution, and 1 g cysteine-HCl. For the fermentation system, each serum bottle contained 90 ml basal medium, 10 ml fecal inoculum, and 1 g arabinoxylan. To evaluate the effect of H_2_ accumulation on fermentation efficiency, the methanogenic inhibitor BES (10 mM) was added to the incubations of Meishan pigs [33]. All incubations were divided into Yorkshire, Meishan, and Meishan + BES groups according to the source of the fecal slurries (*n* = 4). All serum bottles were incubated at 37°C and shaken at 150 rpm for 48 h. Samples were collected at 0, 6, 12, 24, and 48 h after inoculation. The headspace gas was collected to measure H_2_ and CH_4_ production using gas chromatography (Agilent 7890B; Agilent, Palo Alto, CA, USA), as previously described [34]. The pH of the culture medium was determined by a pH meter (Orion Star™ A321, ThermoFisher, USA). Then, the culture medium was sampled and stored at −80°C for further analysis.

### Measurements of nutritional digestibility

The total dietary fiber content in the colonic digesta was quantified using a Total Dietary Fiber Assay Kit (Megazyme, Wicklow, Ireland) according to the manufacturer’s instructions. The crude protein content was measured according to the AOAC procedures. The nutritional digestibility was calculated as follows: CAD_F_ (%) =100 × (1 − (DC_D_ × AIA_F_)/(DC_F_ × AIA_D_)). Where CAD_F_ represents the digestibility of nutrients in the experimental feeds, DC_D_ represents the nutrient content in the colonic digesta, AIA_F_ represents the acid-insoluble ash in the feeds, DC_F_ represents the nutrient content in the feeds, AIA_D_ represents the acid-insoluble ash in the colonic digesta.

### Determination of microbial metabolites

SCFA concentrations in the colonic digesta and fermentation broth were detected by gas chromatography, according to our previous method [35]. Briefly, 0.3 g of the colonic content was fully dissolved in deionized water (1.5 ml) and centrifuged. Next, 1 mL of colonic digestion supernatant or fermentation broth was mixed with 0.2 ml of 25% (w/v) metaphosphoric acid. The mixture was then vortexed for 1 min and centrifuged at 12 000 × *g* for 10 min at 4°C. The supernatant was frozen at −20°C overnight and filtered with a 0.22-μm filter and then used for SCFA measurement. Organic acids (formate, lactate, and succinate) and biogenic amines were quantified using high-performance liquid chromatography (Ultimate3000, Thermo Corporation, MA, USA) following previously published protocols [36].

### DNA extraction and 16S rRNA gene sequencing

Total genomic DNA was extracted from the colonic digesta using a DNA Stool Mini Kit (QIAGEN, Hilden, Germany), according to the manufacturer’s instructions. The concentration of the extracted DNA was detected using a NanoDrop 2000 (Thermo Scientific, USA), and DNA quality was determined using agarose gel electrophoresis. The V3 and V4 regions of the bacterial 16S rRNA gene were amplified by PCR using universal primers 341F (5′-ACTCCTACGGGAGGCAGCAG-3′) and 806R (5′-GGACTACHVGGGTWTCTAAT-3′). The amplicons were purified using a Qubit™ dsDNA BR assay kit (Invitrogen, USA) and were then sequenced on the Illumina Hiseq 2500 platform with a sequencing length of paired-end 300 bp.

16S raw data were quality-filtered and demultiplexed by Quantitative Insights Into Microbial Ecology 2 (QIIME 2) pipeline (version 2020.2) [37]. After quality control, a total of 552 376 high-quality reads were obtained with an average of 46 031 reads per sample. Operational taxonomic units (OTUs) were clustered based on 97% sequence similarity using UPARSE (version 7.1). Taxonomic assignment of the OTUs was generated using the Ribosomal Database Project Classifier against the Silva reference database (version 138). Alpha- and beta-diversity, and PERMANOVA analysis were also analyzed using QIIME 2.

### RNA extraction and metatranscriptome sequencing

Total RNA was extracted from the colonic digesta using the HiPure Stool RNA Kit (MAGEN, Shanghai, China) according to the manufacturer’s instructions. RNA concentration and quality were evaluated using a Fragment Analyzer standard sensitivity RNA kit (AATI, DNF-471) following standard protocols. After the removal of DNA and rRNA, the fragmented RNA was synthesized into double-stranded cDNA. DNA nanoball-based libraries were constructed using the MGIEasy Small RNA Library Prep Kit V2.0 (MGI, Shenzhen, China) according to the manufacturer’s instructions. The libraries were then sequenced using the MGISEQ-2000 platform, which generated paired-end 100-bp sequences.

### Metatranscriptomic analysis

Metatranscriptomic sequencing generated 257.87 Gb (average of 21.49 Gb per sample) of raw data from 12 colonic digesta samples. The SOAPnuke (version 1.5.2) software was used to trim the adapters and remove low-quality sequences from the raw data. Short Oligonucleotide Analysis Package 2 (SOAP2) was used to identify and remove host sequences, and a total of 239.24 Gb (average of 19.94 Gb per sample) of high-quality reads were acquired. High-quality clean reads from each sample were assembled de novo using IDBA TRAN software (version 1.1.3). MetaGeneMark (version 2.10) was used to predict the assembled contigs. A non-redundant data set was constructed using CD-HIT (version 4.6.1) with a sequence identity cutoff of 90%. Finally, 1 181 803 non-redundant genes were obtained from the 12 colonic digesta samples. A summary of the metatranscriptome datasets is provided in Supplementary Table S2.

Taxonomic assignment of the gene catalog for each sample was performed using DIAMOND (version 0.8.23.85), based on the BLASTP procedure against the NCBI-NR database, with an *E* value of 1e-5 as the cut-off. Phylogenetic levels were analyzed using the lowest common ancestor-based algorithm in the MEGAN software (version 5). Protein sequences in the gene catalog were mapped to the CAZyme database using HMMER (version 3.2.1) to annotate the CAZyme profiles. The protein sequences were then matched to the GENES database using Diamond to identify the Kyoto Encyclopedia of Genes and Genomes (KEGG) gene profile. Terminal reductases associated with H_2_ uptake pathways were identified by referring to Greening *et al.* [38], which were determined by DIAMOND searches against respective gene sequences (with an *E*-value threshold of 1e-50, coverage values exceeding 90% and identity values exceeding 40%). The expression level of each gene was calculated using transcripts per million (TPM) method [39], and the abundances of taxa, CAZymes, Kos, and terminal reductases were summarized based on the abundance of annotated genes.

### Microbe quantification

After extraction of total genomic DNA from the colonic digesta and fermentation broth, q real-time PCR was performed to determine the quantities of total bacteria, Bacteroidetes, Firmicutes, *Bacteroides*, *Prevotella*, *Blautia*, *Methanobrevibacter*, *Desulfovibrio*, and hydrogenotrophic functional genes (AcsB, McrA, and AprA). All qPCR assays were performed on an ABI 7300 real-time PCR system (Applied Biosystems, Foster City, CA, USA) using SYBR Green dye (Takara, Kusatsu, Japan). The PCR reaction mixture with a total volume of 20 μl contained 10 μl SYBR fluorescence dye, 2 μl DNA template (20 ng), 0.4 μl each of the forward and reverse primer, 0.4 μl ROX reference dye, and 6.8 μl nuclease-free water. The copy numbers of target microbial groups were calculated through the standard curves constructed with 10-fold serial dilutions of plasmid DNA. The primer sequences for the selected microorganisms and the construction of standard plasmids refer to previous studies, as shown in Supplementary Table S3.

### Measurement of polysaccharide-degrading enzyme activities

The activities of β-xylosidase and β-glucosidase were determined by p-nitrophenyl glycoside-based methods as previously described [40]. The carboxymethyl cellulase and pectinase were measured using a colorimetric kit (Nanjing Jiancheng Biotechnology, Nanjing, China) according to the manufacturer’s instructions.

### Targeted metabolomics for reducing sugar quantification

Targeted metabolomics was performed to quantify the reducing sugars in the colonic content using ultra-performance liquid chromatography coupled with tandem mass spectrometry (UPLC-MS/MS; ACQUITY UPLC Xevo TQ S, Waters Corp., Milford, MA, USA). Briefly, 10 mg freeze-dried contents were weighed into a centrifuge tube and were then added with 25 μl deionized water and 120 μl methanol solution containing internal standard. The mixed solution was centrifuged at 18 000 × *g* at 4°C for 20 min, and 40 μl supernatant was taken into 96-well plates. Then, 40 μl of derivative reagent and 330 μl of 50% methanol solution were added to each well. Finally, 135 μl supernatant was transferred to a new 96-well plate for reducing sugar quantification. For data processing, raw data files generated by UPLC-MS/MS were analyzed using the iMAP platform (v1.0; Metabo-Profile, Shanghai, China).

### Statistical analysis

The Wilcoxon rank-sum test was used to assess significant differences between the two groups, and the unpaired two-tailed Student’s t-test was used to compare differences among multiple groups. All graphs in the current paper were plotted using GraphPad Prism 7.0 software (La Jolla, CA, USA). The results were shown as the means ± standard error of the mean (SEM). A value of *P* < .05 was declared statistically significant. False discovery rate correction by the Benjamini–Hochberg method was performed to reduce the false positive rate for the high amount of microbiome data, and an adjusted *P* value (*q* value) < .05 was regarded as significant.

## Results

### Meishan pigs exhibited higher fiber digestibility and more SCFA production in the colon compared to Yorkshire pigs

**Figure 1:**
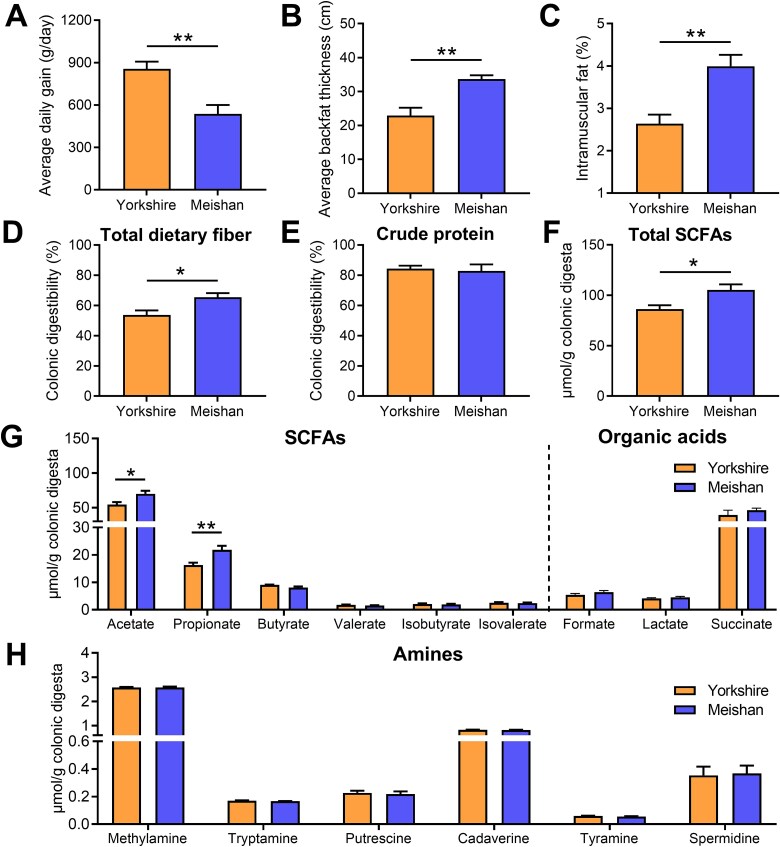
Comparison of fat deposition, colonic nutrient digestibility, and microbial metabolite profiles between Meishan and Yorkshire pigs. (A) Average daily gain. (B) Average backfat thickness. (C) Intramuscular fat content. (D) The digestibility of total dietary fiber in the colonic digesta. (E) The digestibility of crude protein in the colonic digesta. (F) The total SCFA concentration in the colonic digesta. (G) The concentrations of SCFAs and organic acids in the colonic digesta. (H) The concentrations of amines in the colonic digesta. Data are expressed as means ± SEM. The Wilcoxon rank-sum test was used to analyze differences between Yorkshire and Meishan groups (*n* = 6 per group); ^*^P < .05, ^**^P < .01.

In this study, genetically distinct pig breeds (obese-type Meishan and lean-type Yorkshire) were employed as an experimental model and offered the same diet under the same environment for 28 days. The results showed that Meishan pigs had a slower growth rate but a greater capacity for fat deposition, with higher backfat thickness and intramuscular fat content than Yorkshire pigs (Fig. 1A–C). To uncover the inter-breed variability in intestinal fiber degradation, we first measured the colonic digestibility of the main nutrient substrates. Our results showed that the digestibility of total dietary fiber was significantly higher in Meishan pigs than in Yorkshire pigs (Fig. 1D), whereas no difference in crude protein digestibility was observed between the two pig breeds (Fig. 1E). SCFAs and organic acids are the major carbohydrate fermentation products of the gut microbiota and serve as vital energy sources for the host. Nitrogenous compounds such as amino acids are converted by the gut microbiota to produce biogenic amines. Compared to Yorkshire pigs, Meishan pigs showed higher concentrations of total SCFAs, acetate, and propionate in the colon (Fig. 1F and G). There were no differences in the biogenic amine levels in the colonic digesta between the two pig breeds (Fig. 1H). These results indicate that Meishan pigs have a greater ability to ferment plant polysaccharides to SCFAs than Yorkshire pigs.

### Meishan pigs harbored more abundant *Bacteroides* species and H_2_-utilizing methanogens in the colon compared to Yorkshire pigs

**Figure 2:**
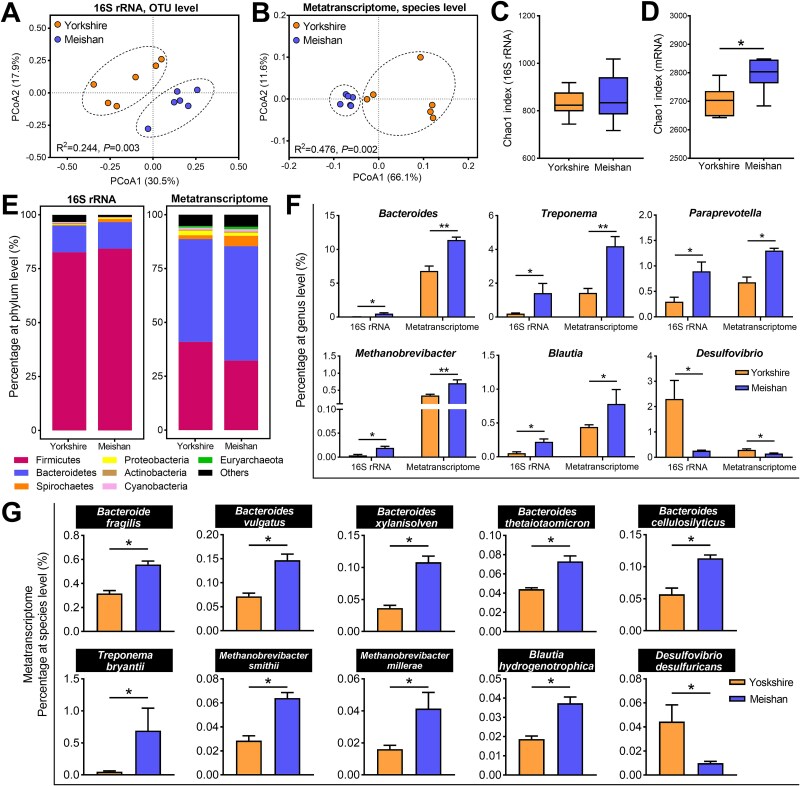
Comparison of colonic microbiota composition between Meishan and Yorkshire pigs based on 16S rRNA gene sequencing and metatranscriptomics. (A) PCoA profile of colonic bacterial community based on the Bray-Curtis distance at the OTU level (PERMANOVA, *P* = .003, *R*^2^ = 0.244). (B) PCoA profile of colonic bacterial community based on the Bray-Curtis distance at the metatranscriptomic species level (PERMANOVA, *P* = .002, *R*^2^ = 0.476). (C) Chao 1 diversity analysis based on 16S rRNA. (D) Chao 1 diversity analysis based on metatranscriptomics. (E) The dominant microbial phyla in Meishan and Yorkshire pigs based on 16S rRNA and metatranscriptomics. (F) Differential microbial genera between Meishan and Yorkshire pigs based on 16S rRNA and metatranscriptomics. (G) The representative significantly differential polysaccharide-degrading and H_2_-utilizing species between the two breed pigs based on metatranscriptomic data. Data are expressed as means ± SEM. The Wilcoxon rank-sum test was used to analyze differences between Yorkshire and Meishan groups (*n* = 6 per group); ^*^adjusted *P* < .05, ^**^adjusted *P* < .01.

16S rRNA gene sequencing reveals the microbial community at the DNA level, and metatranscriptome accurately reflects the physiologically active microbial community and its function at the RNA level. In the present study, 16S rRNA gene sequencing and metatranscriptomics were performed to compare the colonic microbial composition of two pig breeds. Principal coordinate analysis (PCoA) based on 16S rRNA and metatranscriptome showed clear segregation of the microbiota structure between Yorkshire and Meishan pigs (Fig. 2A and B). Analysis of microbial diversity revealed that Meishan pigs had a higher Chao1 index than that of Yorkshire pigs at the RNA level (Fig. 2C and D). The dominant microbial phyla are shown in Fig. 2E. There was no significant difference in the relative abundances of Firmicutes and Bacteroidetes between the two pig breeds, whereas higher relative abundances of Spirochaetes and Euryarchaeota were observed in Meishan pigs than in Yorkshire pigs based on metatranscriptome (Supplementary Fig. S1). At the genus level, we found that the abundances of several genera associated with polysaccharide degradation, including *Bacteroides*, *Treponema*, and *Paraprevotella*, were significantly higher in Meishan pigs than in Yorkshire pigs at the DNA and the RNA level (Fig. 2F). A total of 425 active microbial species have been identified in Yorkshire and Meishan pigs based on metatranscriptome data (Supplementary Fig. S2). Specifically, many species of *Bacteroides* were significantly enriched in Meishan pigs, including *Bacteroides fragilis*, *Bacteroides vulgatus*, *Bacteroides xylanisolven*, *Bacteroides thetaiotaomicron*, and *Bacteroides cellulosilyticus* (Fig. 2G).

Moreover, several microbes related to hydrogen utilization displayed marked differences between Meishan and Yorkshire pigs. The relative abundances of methanogenic *Methanobrevibacter* and acetogenic *Blautia* were higher in Meishan pigs at the DNA and the RNA level, whereas the abundance of the SRB *Desulfovibrio* was higher in Yorkshire pigs (Fig. 2F). At the species level, Meishan pigs had higher abundances of *Methanobrevibacter smithii, Methanobrevibacter millerae*, and *Blautia hydrogenotrophica* and a lower abundance of *Desulfovibrio desulfuricans* than Yorkshire pigs (Fig. 2G). Correlation analysis showed that the relative abundances of *Bacteroides*, *Methanobrevibacter*, *Paraprevotella*, and *Treponema* positively correlated with intramuscular fat content, total fiber digestibility, and SCFA levels (Supplementary Fig. S3). Therefore, these results suggest that the colonic microbiota community differed between Meishan and Yorkshire pigs, which is related to hydrogen utilization and polysaccharide breakdown.

### CAZyme distribution in the colonic microbiome of pigs

To determine the capacity of the colonic microbiome for plant polysaccharide degradation, we screened for CAZymes in assembled metatranscriptomic contigs and determined their phylogenetic distributions. Overall, 11 242 CAZyme-encoding genes were retrieved and grouped into 196 CAZyme families, including 101 glycoside hydrolase (GH), 24 glycosyl transferase, 13 polysaccharide lyase (PL), 13 carbohydrate esterase (CE), 41 carbohydrate-binding module, and 4 auxiliary activity families. Based on the abundance of TPM, GH had the highest relative abundance among the six CAZyme classes, accounting for 51.5% of the total CAZyme (Supplementary Fig. S4A). Bacteroidetes and Firmicutes were the major phyla assigned to the CAZyme genes (Supplementary Fig. S4B).

CAZymes were classified into four categories based on the substrate specificity of GH, PL, and CE, targeting the degradation of pectin, cellulose, starch, and arabinoxylan. Among the 30 enzymes screened, arabinoxylan-degrading enzymes with the highest relative abundances (16.1% of total CAZymes) were mainly phylogenetically assigned to Bacteroidetes (*Prevotella*, 32.5%; *Bacteroides*, 23.0%), Firmicutes (*Ruminococcus*, 9.3%), and Spirochaetes (*Treponema*, 1.5%) (Supplementary Fig. S5). Enzymes specifically involved in starch degradation accounted for 6.7% of total CAZyme expression, which was primarily assigned to *Prevotella* (55.7%) and *Bacteroides* (10.7%) at the genus level (Supplementary Fig. S5). Similarly, the pectin-targeting enzymes, representing 4.16% of total CAZymes, were mainly associated with the Bacteroidetes (*Prevotella*, 50.0%; *Bacteroides*, 12.2%) (Supplementary Fig. S5). However, cellulose-degrading enzymes (5.0% of total CAZymes) mostly originated from Firmicutes (*Ruminococcus*, 36.3%) (Supplementary Fig. S5). These data reveal that members of *Prevotella*, *Bacteroides*, and *Ruminococcus* are predominant plant polysaccharide degraders in pig colon.

### Arabinoxylan-degrading CAZymes were more abundant in the Meishan pigs compared to Yorkshire pigs

**Figure 3:**
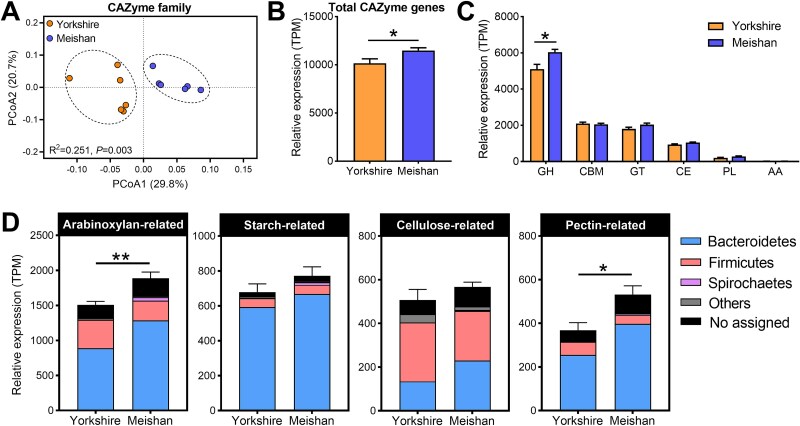
Comparison of the microbial CAZyme profiles between Meishan and Yorkshire pigs. (A) PCoA profile of the CAZyme family (PERMANOVA, *P* < .003, *R*^2^ = 0.251). (B) The total expression levels of CAZyme genes in Meishan and Yorkshire pigs. (C) The relative expression levels of the CAZymes gene families in Meishan and Yorkshire pigs. (D) The relative expression levels of CAZyme gene families involved in the degradation of arabinoxylan, starch, cellulose, and pectin in Meishan and Yorkshire pigs. Data are expressed as means ± SEM. The Wilcoxon rank-sum test was used to analyze differences between Yorkshire and Meishan groups (*n* = 6 per group); ^*^P < .05, ^**^P < .01.

**Figure 4:**
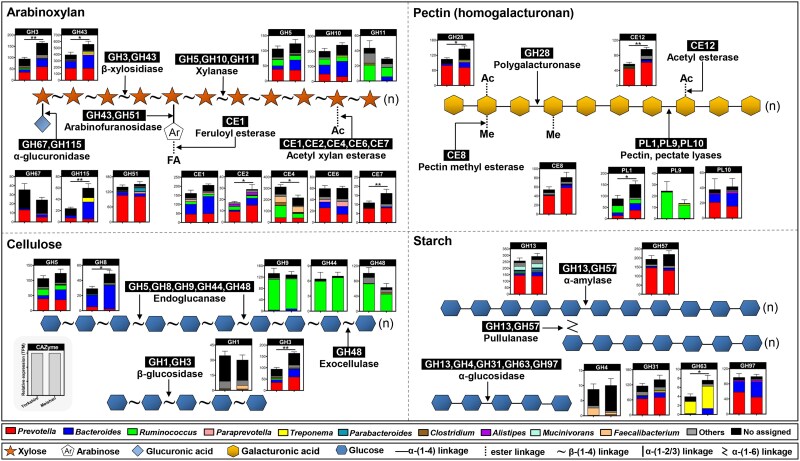
Comparison of the expression levels and distribution of CAZyme families targeting different carbohydrate substrates in the colonic microbiome between Meishan and Yorkshire pigs. The phylogenetic distribution of CAZyme families assigned to the identified genus, no assigned means there is no specific taxonomic information at the genus level. Data are expressed as means ± SEM. The Wilcoxon rank-sum test was used to analyze differences between Yorkshire and Meishan groups (*n* = 6 per group); ^*^P < .05, ^**^P < .01.

Next, we compared the CAZyme profiles of the two pig breeds. PCoA plots based on Bray–Curtis dissimilarity at the CAZyme family level clearly showed different clusters between the Yorkshire and Meishan samples (Fig. 3A). Meishan pigs showed higher expression levels of total CAZyme genes and the GH subfamily than Yorkshire pigs (Fig. 3B and C). The expression levels of arabinoxylan- and pectin-degrading CAZymes were higher in Meishan pigs than in Yorkshire pigs (Fig. 3D). Employing these 30 CAZyme families targeting different substrates and their top 10 assigned genera, a pattern diagram of plant polysaccharide degradation by colonic microbes was constructed (Fig. 4). We found that the enzymes contributing to arabinoxylan degradation (GH43, GH3, GH115, CE2, and CE7) and pectin degradation (PL1, GH28, and CE12) were significantly higher in Meishan pigs than in Yorkshire pigs (Fig. 4). GH43 (β-xylosidase), as the most represented family involved in the breakdown of arabinoxylan, was mainly affiliated to *Bacteroides*, *Prevotella*, and *Treponema* (Fig. 4). The key pectin-degrading enzyme PL1 (pectin lyase) was primarily encoded by *Prevotella*, *Ruminococcus*, and *Bacteroides* (Fig. 4). Furthermore, we focused on the phylogenetic distribution of CAZymes highly expressed in Meishan pigs at the species level, and several pivotal species belonging to *Bacteroides* (e.g. *B. cellulosilyticus* and *B. vulgatus*) were identified (Supplementary Fig. S6). Thus, Meishan pigs harbored more abundant CAZymes in the colonic microbiome than Yorkshire pigs, which mainly contributed to the degradation of arabinoxylan and pectin.

### Enzymes in xylose utilization and SCFA production pathways were significantly enriched in Meishan pigs compared to Yorkshire pigs

**Figure 5:**
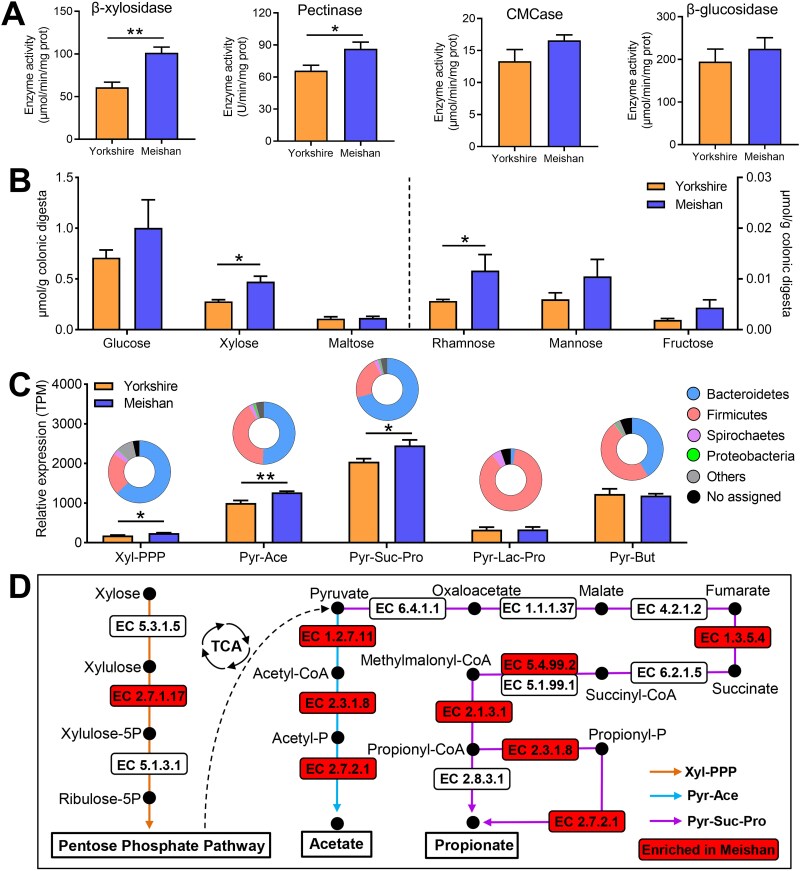
Comparison of the metabolic pathways and enzymes involved in xylose utilization and SCFA production between Meishan and Yorkshire pigs. (A) The activities of specific enzymes targeting different plant polysaccharides. (B) The concentration of reducing sugars in the colonic digesta. (C) The expression levels and distribution of the KEGG genes involved in xylose utilization and SCFA production pathways; and pie charts show the phylogenetic distribution of the related pathways at the phylum level. Xyl-PPP: catabolic pathway of xylose into pentose phosphate pathway, Pyr-Ace: acetate synthesis from pyruvate, Pyr-Suc-Pro: pyruvate fermentation to propionate via succinate, Pyr-Lac-Pro: pyruvate fermentation to propionate via lactate, Pyr-But: butyrate synthesis from pyruvate. (D) The metabolic pathways and changed enzymes that xylose is metabolized by microbiota to produce acetate and propionate. Data are expressed as means ± SEM. The Wilcoxon rank-sum test was used to analyze differences between Yorkshire and Meishan groups (*n* = 6 per group). ^*^P < .05, ^**^P < .01.

To further validate the distinct capacities of the two pig breeds for plant polysaccharide degradation revealed by the metatranscriptomes, we detected key enzyme activities and reducing sugar content in the colonic digesta. Compared with Yorkshire pigs, the activities of enzymes targeting linkages in arabinoxylan (β-xylosidase) and pectin (pectinase) were higher in Meishan pigs, while others related to cellulose degradation were not significant (Fig. 5A). Furthermore, higher levels of xylose and rhamnose were observed in Meishan pigs (Fig. 5B). By mapping KEGG orthologous genes, we screened genes encoding enzymes involved in the xylose utilization and SCFA production pathways. Higher expression levels of enzymes involved in xylose catabolism, acetate synthesis, and propionate synthesis (succinate pathway) were observed in Meishan pigs than in Yorkshire pigs (Fig. 5C and D, and Supplementary Fig. S7). These results demonstrate that compared to Yorkshire pigs, Meishan pigs possess a stronger ability to degrade arabinoxylan and produce acetate and propionate.

### Hydrogenotrophic methanogenesis and acetogenesis pathways were significantly enriched in Meishan pigs compared to Yorkshire pigs

**Figure 6:**
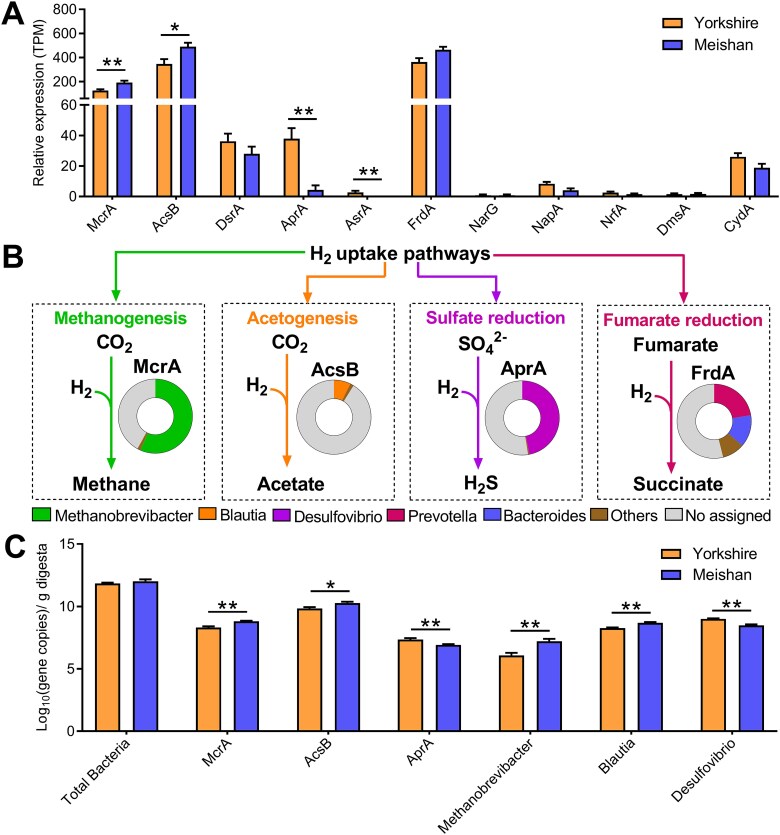
Comparison of the H_2_ disposal pathways in the colonic microbiome between Meishan and Yorkshire pigs. (A) The relative expression levels of the terminal reductases representing the different H_2_ uptake pathways between Yorkshire and Meishan pigs. McrA, methyl-CoM reductase; AcsB, acetyl-CoA synthase; DsrA, dissimilatory sulfite reductase; AprA, adenylylsulfate reductase; AsrA, alternative sulfite reductase; FrdA fumarate reductase; NarG, dissimilatory nitrate reductase; NapA, periplasmic nitrate reductase; NrfA, ammonia-forming nitrite reductase; DmsA, DMSO and TMAO reductase; CydA, cytochrome bd oxidase. (B) The dominant pathways and microorganisms responsible for colonic H_2_ uptake in pigs. The pie charts show the phylogenetic distribution of the reductases at the genus level, no assigned means there is no specific taxonomic information at the genus level. (C) The copy numbers of total bacterial 16S rRNA genes, representative H_2_-utilizing functional genes, and microbial genera in the colonic digesta of Yorkshire and Meishan pigs. Data are expressed as means ± SEM. The Wilcoxon rank-sum test was used to analyze differences between Yorkshire and Meishan groups (*n* = 6 per group); ^*^P < .05, ^**^P < .01.

Considering the significant differences observed in the hydrogenotrophic species between the two pig breeds, we speculate that they have distinct hydrogen disposal pathways. We then screened for functional genes representing different H_2_ uptake pathways in the assembled metatranscriptomic contigs (McrA: methanogenesis; AcsB: acetogenesis; DsrA, AprA, AsrA: sulfate reduction; FrdA: fumarate reduction; CydA: aerobic respiration; NarG, NapA, NrfA: nitrate ammonification; and DmsA: DMSO and TMAO reduction). These data showed that acetogenesis, methanogenesis, sulfate reduction, and fumarate reduction were the predominant pathways for H_2_ disposal, suggesting that H_2_ was mainly converted to acetate, CH_4_, H_2_S, and succinate in the pig colon (Fig. 6A and B). Notably, Meishan pigs had higher expression levels of McrA and AcsB and lower expression levels of AprA and AsrA than Yorkshire pigs (Fig. 6A). McrA and AprA originated mainly from *Methanobrevibacter* and *Desulfovibrio*, respectively (Fig. 6B). FrdA was primarily encoded by *Prevotella* and *Bacteroides* and AcsB was partly assigned to *Blautia* (Fig. 6B). Consistently, qPCR results confirmed that Meishan pigs had higher copy numbers of McrA, AcsB, *Methanobrevibacter* and *Blautia,* and lower copy numbers of AprA and *Desulfovibrio* than Yorkshire pigs (Fig. 6C).

### Inhibition of methanogens resulted in hydrogen accumulation and restricted arabinoxylan degradation in Meishan pigs

**Figure 7:**
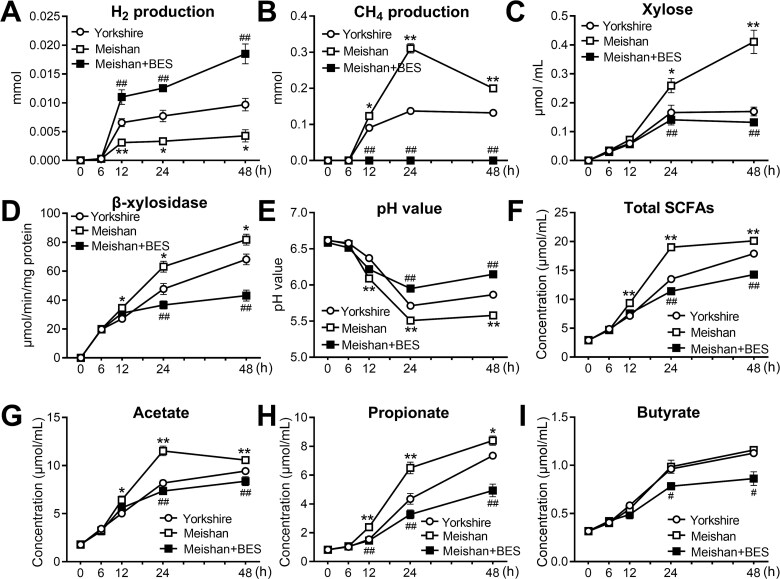
Comparison of the capacity for arabinoxylan degradation and H_2_ disposal of the fecal microbiota in Meishan and Yorkshire pigs using an *in vitro* batch incubation model. (A) H_2_ and (B) CH_4_ production during the incubation of arabinoxylan by fecal inoculum from Yorkshire and Meishan pigs. (C) Xylose levels during the fermentation. (D) The activities of β-xylosidase during the fermentation. (E) pH value during the fermentation. (F–I) The concentrations of SCFAs during the fermentation. Data are expressed as means ± SEM. An unpaired two-tailed Student’s t-test was used to assess differences among groups (*n* = 4 per group); ^*^P < .05, ^**^P < .01 compared to Yorkshire group; ^#^*P* < .05, ^##^*P* < .01 compared to Meishan + BES group.

To explore whether the hydrogenotrophic methanogenesis represents a differential factor driving inter-breed variation in arabinoxylan degradation, *in vitro* batch incubations were performed. The fecal microbiota of Meishan and Yorkshire pigs was used as the inoculum, and arabinoxylan was selected as the sole carbon source for *in vitro* incubations. Here, 2-bromoethanesulfonate (BES), a methanogen inhibitor, was used to block the conversion of H_2_ to CH_4_ and assess the effect of methanogens on the bacterial degradation efficiency of arabinoxylan. We observed that the H_2_ level was significantly lower in the Meishan group than in the Yorkshire group 12 h after inoculation, whereas it showed a clear increase when BES was added to the incubations of Meishan pigs (Fig. 7A). Additionally, a higher CH_4_ level was observed in the Meishan group than in the Yorkshire group (Fig. 7B). No CH_4_ was detected throughout the incubation process when BES was added to the incubations of Meishan pigs (Fig. 7B), verifying that this compound effectively inhibited CH_4_ production and caused the accumulation of H_2_. The β-xylosidase activity and xylose concentration were detected to evaluate the arabinoxylan degrading status. Significantly higher levels of xylose and β-xylosidase were observed during fermentation in Meishan group compared with the Yorkshire group, while these showed an obvious decline in the Meishan pigs when BES was added (Fig. 7C and D). The pH and SCFA levels were determined to reflect the acid-producing capacity. Compared to the Yorkshire group, the Meishan group exhibited a lower pH value with higher concentrations of total SCFAs, acetate, and propionate 12 h after inoculation (Fig. 7E–I), which further confirmed that Meishan pigs harbored a stronger capacity for arabinoxylan degradation than Yorkshire pigs. In contrast, BES supplementation significantly decreased the concentration of SCFAs in Meishan pigs (Fig. 7E–I). These *in vitro* results suggest that hydrogenotrophic methanogens serve as an important deterministic factor driving arabinoxylan degradation.

### Hydrogenotrophic methanogens strongly contributed to hydrogen disposal and facilitated arabinoxylan degradation by *Bacteroides*

**Figure 8:**
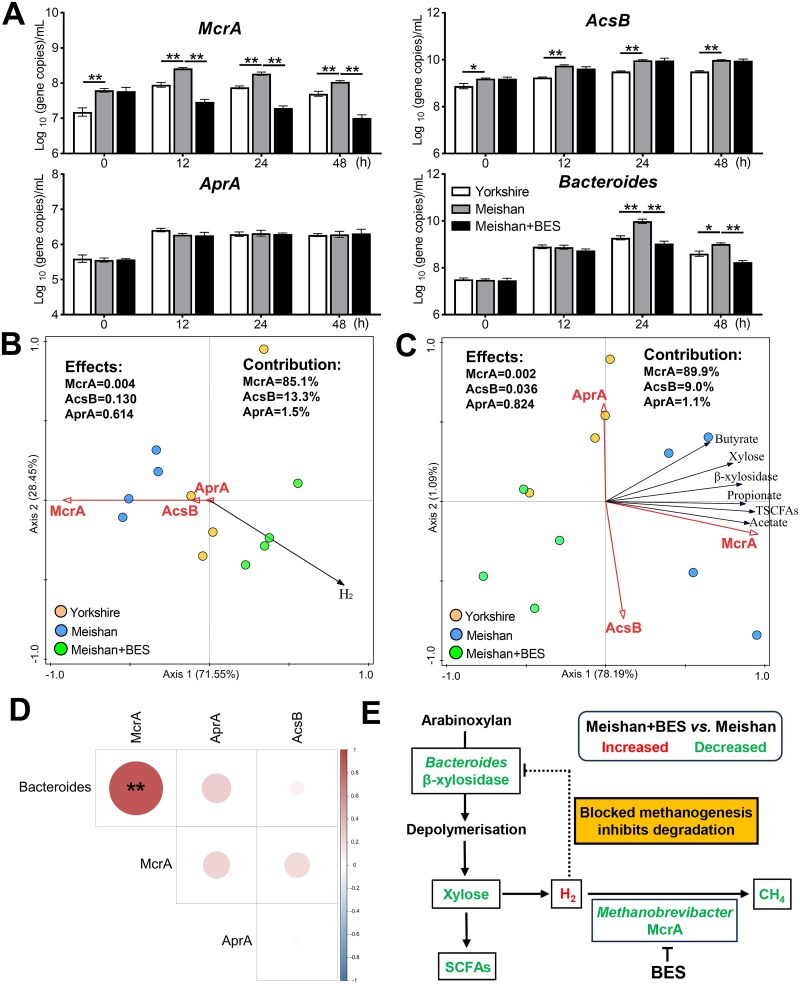
Evaluation of the effects of hydrogenotrophic methanogenesis on H_2_ disposal and arabinoxylan degradation during *in vitro* incubation. (A) qPCR measurement of quantities of hydrogenotrophic functional groups and *Bacteroides* during fermentation among three groups. (B) RDA plots of H_2_ levels and three hydrogenotrophic functional groups at 24 h of fermentation. (C) RDA plots of fermentation parameters and three hydrogenotrophic functional groups at 24 h of fermentation. (D) Correlation analyzes of copy numbers of *Bacteroides* and three hydrogenotrophic functional groups at 24 h of inoculation. (E) The schematic model shows that blocked methanogenesis inhibits arabinoxylan degradation by *Bacteroides* species via increasing H_2_ partial pressure. Data are expressed as means ± SEM. An unpaired two-tailed Student’s t-test and Spearman’s method were used to assess differences among groups (*n* = 4 per group); ^*^P < .05, ^**^P < .01.

To evaluate the abundance of methanogens in the overall microbiota, we determined the quantities of hydrogenotrophic functional groups (McrA, methanogens; AcsB, acetogens; AprA, SRB), representative microbial phyla and genera during *in vitro* fermentation using qPCR. Remarkably, the Meishan group exhibited significantly higher copy numbers of McrA and AcsB at baseline (0 h) and throughout the fermentation period than the Yorkshire group, whereas no difference in the quantity of AprA was observed between the Meishan and Yorkshire groups during fermentation (Fig. 8A). Adding BES to the incubations of Meishan pigs significantly decreased the copy numbers of McrA after 12 h of inoculation; however, this did not affect the copy numbers of AcsB or AprA (Fig. 8A). The representative H_2_-utilizing genera showed similar trends among the three groups (Supplementary Fig. S8). The quantities of total bacteria, Bacteroidetes, and *Bacteroides* were higher in the Meishan group than in the Yorkshire group 24 h after inoculation, whereas there was a significant decrease in the Meishan group when BES was added to the incubations (Fig. 8A and Supplementary Fig. S8).

Redundancy analysis (RDA) of H_2_ levels and fermentation parameters across samples at 24 h of inoculation was used to assess the relative contributions of the different hydrogenotrophic functional groups to H_2_ disposal and arabinoxylan degradation. Compared with acetogens and SRB, methanogens significantly affected the H_2_ levels and had the largest contribution to H_2_ disposal (contribution = 85.1%) (Fig. 8B). Furthermore, methanogens and acetogens significantly impacted the fermentation parameters of arabinoxylan, whereas SRB had no significant effect on arabinoxylan degradation (Fig. 8C). Among these, methanogens had the largest contribution to the fermentation degree of arabinoxylan (contribution = 89.9%), followed by acetogens (contribution = 9.0%) and SRB (contribution = 1.1%). Spearman’s correlation analysis further showed that *Bacteroides* numbers were positively correlated with McrA, and they were both positively correlated with the levels of β-xylosidase, xylose, and SCFAs at 24 h of inoculation (Fig. 8D and Supplementary Fig. S8). As shown in Fig. 8E, a schematic model shows that blocked methanogenesis led to the accumulation of H_2_ which restricted arabinoxylan degradation by *Bacteroides* species and finally reduced the SCFA yield.

## Discussion

By employing genetically distinct pig breeds (obese-type Meishan and lean-type Yorkshire), the present study uncovered inter-breed differences in gut microbiota composition and functional activities, which were characterized by higher abundances of polysaccharide-degrading bacteria (*Bacteroides*, *Treponema*, and *Paraprevotella*) and hydrogenotrophic microbes (*Methanobrevibacter* and *Blautia*), together with greater arabinoxylan degradation activity and enhanced methanogenesis pathways in the Meishan gut microbiome. *In vitro* fermentation experiments further revealed that the high abundance of methanogens in Meishan pigs maintained lower hydrogen levels through methanogenesis, thus facilitating arabinoxylan degradation by *Bacteroides*, and finally leading to increased SCFA production. The findings demonstrated that as compared with the lean-type Yorkshire pigs, the obese-type Meishan pigs exhibited greater ability of fiber degradation, which is associated with the stronger methanogenesis pathway.

### *Bacteroides*-dominant microbiome contributes to enhanced arabinoxylan degradation in obese-type Meishan pigs

*Bacteroides* harbor relatively large genomes that encode a broad repertoire of CAZymes and are the most efficient polysaccharide degraders in the human gut [1, 41]. The present study showed that the obese Meishan pigs have a higher abundance of *Bacteroides* than Yorkshire pigs, which is consistent with the results of our previous study based on 16S rRNA gene amplicon sequencing [28]. Notably, at the species level, *B. fragilis*, *B. vulgatus*, *B. xylanisolven*, *B. thetaiotaomicron*, and *B. cellulosilyticus* were significantly enriched in Meishan pigs, all of which possess xylanolytic activity involved in the degradation of arabinoxylan [42]. Other members associated with polysaccharide degradation, such as *Treponema* and *Paraprevotella*, were also more abundant in Meishan pigs. Although *Treponema* rarely colonizes the human gut, it is widely present in the pig gut and has shown great potential for degrading plant polysaccharides based on metagenomic studies [43]. A recent study has reported that several species belonging to *Bacteroides* and *Treponema* are positively correlated with weight gain in pigs [44].

Members of the gut microbiota differ in their ability to degrade different types of plant polysaccharides depending on the CAZymes they encode [45]. We further compared the CAZymes targeting the main plant polysaccharides (arabinoxylan, pectin, cellulose, and starch) present in the colonic microbiome of pig breeds. Similar to a previous discovery in the human gut microbiome [1], Bacteroidetes members encode more CAZyme genes than other phyla in the pig gut microbiome. At the genus level, the genes encoding for CAZymes were mainly assigned to *Prevotella*, *Bacteroides*, and *Ruminococcus*, but rarely originated from *Treponema* and *Paraprevotella*. Notably, Bacteroidetes (mostly *Prevotella* and *Bacteroides*) mainly contributed to arabinoxylan-related CAZymes, whereas Firmicutes (mostly *Ruminococcus*) mainly contributed to cellulose-related CAZymes. Arabinoxylan is the most common hemicellulose rich in maize and wheat and has been shown to shape the gut microbial community and has multiple benefits for host health [46]. CAZymes for arabinoxylan degradation were also more abundant than those for other substrates, suggesting a functional adaptation of the gut microbiome in the porcine gut.

Arabinoxylan-related CAZymes (e.g. GH3 and GH43) were highly enriched in Meishan pigs relative to Yorkshire pigs and were largely contributed by *Bacteroides* members. Many *Bacteroides* species express xylanolytic activity, whereas very few species have cellulose-degrading activity in the human gut [47]. For example, *B. xylanisolven* can degrade arabinoxylan but does not exhibit the ability to use cellulose and starch [48]. This may explain the higher abundances of *Bacteroides* species and CAZymes that target arabinoxylan in Meishan pigs. Moreover, increased β-xylosidase activities and xylose contents in Meishan pigs further verified the breed-related variability in microbial arabinoxylan degradation.

### Superior capacity for arabinoxylan degradation in obese-type Meishan pigs is driven by hydrogenotrophic methanogenesis

In the present study, by integrating 16S rRNA sequencing, metatranscriptomic, and qPCR analysis, we identified significantly higher numbers of methanogens (e.g. *Methanobrevibacter*) and acetogens (e.g. *Blautia*) in Meishan pigs. Methanogens possess a far lower threshold of hydrogen concentration and are more thermodynamically favorable than acetogens [6]. Multiple evidence has demonstrated that methanogens can outcompete acetogens for hydrogen and release more energy in the gut [49, 50], suggesting that methanogenesis is a more effective hydrogen disposal pathway than acetogenesis. Large volumes of hydrogen are produced in the gut via microbial polysaccharide degradation pathways. Polysaccharide-degrading bacteria, including *Ruminococcus* and *Bacteroides*, have been reported as major producers of hydrogen in the human gut [51]. Hydrogenotrophic microbes play an essential role in the maintenance of hydrogen partial pressure in gut ecosystem [52].

Hydrogenotrophic methanogens possibly share synergistic metabolic interactions with polysaccharide-degrading *Bacteroide* species [9–11]. *M. smithii* is the most abundant methanogenic *archaeal* species in the human gut and is capable of converting H_2_/CO_2_ or formate into CH_4_ [53]. Based on genomic evidence, investigators have proposed that the capacity of *M. smithii* to consume diverse bacterial end-products of fermentation may confer greater flexibility to form syntrophic relationships with the prominent saccharolytic bacterium *B. thetaiotaomicron* [54]. Co-culture experiments have verified that *B. thetaiotaomicron* fermentation products sufficiently support *M. smithii* growth and the metabolic efficiency of *B. thetaiotaomicron* is greater in the presence of *M. smithii* or in the absence of hydrogen [9]. The methanogen-driven synergistic metabolic interactions are also reported with other bacteria, such as *Ruminococcus* [55] and *Christensenella* [56]. In the present study, *Bacteroides* and hydrogenotrophic methanogens co-existed at high levels in the obese Meishan gut and both were positively correlated with fiber digestibility. Thus, we speculated that the superior ability of dietary fiber utilization in the obese Meishan pigs is linked with the interplay between methanogens and *Bacteroides*. Nevertheless, whether the superior ability of dietary fiber utilization links to the obesity warrants further research.

Using *in vitro* experiments, we validated that the gut microbiome of Meishan pigs efficiently decreased hydrogen accumulation through increased methanogenesis and exhibited greater arabinoxylan degradation capability. In contrast, blocking methanogenesis by BES resulted in hydrogen accumulation and reduced the amounts of total bacteria and *Bacteroides*, thus impairing the degradation efficiency of arabinoxylan. These findings demonstrate that the greater capacity for arabinoxylan degradation in Meishan pigs is associated with increased methanogenesis, which could maintain a lower hydrogen level that allowed for more favorable arabinoxylan degradation by *Bacteroides* species. Similar to our observations, previous *in vitro* studies have shown that methanogen inhibitor addition leads to a high partial pressure of hydrogen and decreased fermentation efficiency [57]. However, we found that the other hydrogenotrophic groups, acetogens, and SRB, did not change when methanogens were inhibited, which suggests that their competition for hydrogen is complex and environmentally dependent. A recent study has revealed that the three hydrogenotrophic groups coexist in the gut and do not necessarily compete for hydrogen [58].

### Is the increased hydrogenotrophic methanogenesis linked with obesity phenotype?

Employing genetically lean and obese breed pigs, this study demonstrated that the obese-type pigs have greater ability of dietary fiber utilization than the lean-type pigs, which is linked with microbial hydrogenotrophy. In soil-feeding termites, hydrogenotrophic methanogenesis was reported to have a role in microbial energy harvesting, with the termites maximizing energy extraction from recalcitrant organic matter in the soil via H_2_-utilizing methanogenesis [59]. Several studies in humans and animals have revealed the relevance of methanogens in host energy homeostasis [11, 21, 60]. Early metagenomic studies revealed that the gut microbiome of genetically obese mice harbors a higher abundance of methanogens and contains more abundant genes involved in polysaccharide degradation than lean controls [60]. Further studies in humans reported a significantly higher level of methanogenic archaea in obese individuals than in normal-weight counterparts [21]. Studies on gnotobiotic mice have suggested that *M. smithii*–*B. thetaiotaomicron* co-colonization produces a significant increase in SCFA production and host adiposity compared to mono-associated *B. thetaiotaomicron* [11]. These investigations suggest that hydrogenotrophic methanogenesis is an important mechanism for increasing SCFA production in obese individuals. As the major microbial metabolites, SCFAs, especially acetate and propionate, are absorbed by the host as energy sources [61]. However, it is still unclear whether the high level of SCFA production in the gut is linked to obesity. In humans, it is generally regarded that obese individuals are linked with high-fat and low-dietary fiber diets. In the present study, the obese-type Meishan pigs showed greater ability of dietary fiber utilization with higher levels of SCFAs, notably acetate and propionate, coupled with stronger microbial hydrogenotrophy, than the lean-type Yorkshire pigs. Nevertheless, further studies are warranted to dissect the role of gut microbial hydrogenotrophy especially methanogenesis in obesity.

It should be noted that since the present study focuses on pigs of different breeds at the same physiological stage, due to the intrinsic variation in inter-breed development, potential confounding factors, such as age, body weight, and genetics, may be underrepresented. This may be solved by future design employing age-matched pigs to explore the mechanisms of gut microbiome and fiber degradation.

While this study uncovers the relationship between hydrogenotrophic methanogenesis and fiber degradation in pigs of different breeds, it has some limitations. To minimize the use of local breed pigs, the study employed a limited number of replicates, which may have reduced statistical power. Although rigorous statistical analyzes were performed, future studies with a larger sample size could increase statistical robustness. Additionally, the study only included two pig breeds (Meishan and Yorkshire) raising questions about the generalizability of the findings to other pig breeds. Further research is needed to determine whether the observed microbial functionality is common across pigs. Moreover, this study did not explore whether hydrogenotrophic methanogenesis is mechanistically linked to obesity, an aspect that warrants future investigation.

## Conclusions

Employing obese breed Meishan pigs and lean breed Yorkshire pigs, the present study uncovers the inter-breed difference in arabinoxylan utilization, which is tightly linked to the capacity of methanogenesis and hydrogen removal in the colon. Namely, a higher methanogenesis may promote arabinoxylan utilization and SCFA production. These findings highlight hydrogenotrophic methanogenesis as an important differential factor contributing to inter-breed variation in intestinal polysaccharide degradation. This study may also provide insights into the relevance of gut microbial hydrogenotrophy in obesity development and the connection between dietary fiber utilization and the obese phenotype.

## Supplementary Material

Supplementary_Information_ycaf043

## Data Availability

All raw reads generated in this study (including 16S rRNA and metatranscriptomic sequencing) have been deposited in the NCBI Sequence Read Archive (SRA) database under accession numbers PRJNA1154426 and PRJNA1129603.
