# Supplementary material for: Superior ability of dietary fiber utilization in obese breed pigs linked to gut microbial hydrogenotrophy
Source: ISME Commun. 2025 Mar 9;5(1):ycaf043. doi: 10.1093/ismeco/ycaf043 (PMC11937827; doi:10.1093/ismeco/ycaf043)
Supplement: Supplementary_Information_ycaf043 [file supplementary_information_ycaf043.pdf]

Supplementary Information

Superior ability of dietary fiber utilization in obese breed pigs linked to gut microbial hydrogenotrophy

Xuan Li[1,2,#], Chunlong Mu[3,#], Haiqin Wu[1,2], Erwin G. Zoetendal[1,2,4], Ruihua Huang[1,2], Kaifan Yu[1,2], Weiyun Zhu[1,2,*]

[1]Laboratory of Gastrointestinal Microbiology, Jiangsu Key Laboratory of Gastrointestinal Nutrition and Animal Health, College of Animal Science and Technology, Nanjing Agricultural University, Nanjing 210095, China

[2]National Center for International Research on Animal Gut Nutrition, Nanjing Agricultural University, Nanjing 210095, China

[3]Department of Biochemistry and Molecular Biology, Cumming School of Medicine, University of Calgary, Calgary, Alberta, Canada

[4]Laboratory of Microbiology, Wageningen University & Research, Wageningen, Netherlands

[#]Xuan Li and Chunlong Mu contributed equally to this work.

***Corresponding author:** Weiyun Zhu, College of Animal Science & Technology, Nanjing Agricultural University, No.1 Weigang Road, Nanjing, Jiangsu, 210095, China

E-mail: zhuweiyun@njau.edu.cn

Table S1. Composition and nutrient levels of trial diet

Ingredients	Percentage (%)	Nutrient levels	Percentage (%)
Corn	71.21	ME (MJ/kg)	12.38
Soybean meal	16.65	Crude protein	14.00
Wheat bran	8.21	Ca	0.49
Wheat middling and reddog	1.00	Total P	0.45
Lys	0.61	Available P	0.22
Met	0.02	Lys	1.10
$CaHPO_3$	0.50		
Rock powder	0.80		
Premix[a]	1.00		
Total	100		

Note: [a]Supplied the following per kg of diet: vitamin A, 10,800 IU; vitamin D3, 4,000 IU; vitamin E, 40 IU; vitamin K3, 4 mg; vitamin B1, 6 mg; vitamin B2, 12 mg; vitamin B6, 6 mg; vitamin B12, 0.05 mg; biotin, 0.2 mg; folic acid, 2 mg; niacin, 50 mg; D-calcium pantothenate, 25 mg; Fe, 100 mg as ferrous sulfate; Cu, 150 mg as copper sulfate; Mn, 40 mg as manganese oxide; Zn, 100 mg as zinc oxide; I, 0.5 mg as potassium iodide; and Se, 0.3 mg as sodium selenite.

Table S2. Summary of metatranscriptome dataset

Sample ID	Raw data (Mbp)	Clean data (Mbp)	Contig Number	Assembly Length (bp)	N50 (bp)	N90 (bp)	Average Size (bp)	Mapping Rate (%)	Denovogenes
M1	21489	20164	106456	50013321	446	318	469	20.07	99446
M2	21489	20253	242856	127410492	512	325	524	34.24	245047
M3	21489	20558	234782	124088604	518	326	528	30.44	243581
M4	21489	20584	188105	94530856	487	323	502	25.41	183494
M5	21489	19320	227733	117028965	499	324	513	33.33	231692
M6	21489	18434	198626	106465756	526	326	536	31.57	203818
Y1	21489	20273	156671	81582463	509	325	520	22.85	157493
Y2	21489	18810	179215	96139827	529	327	536	27.45	188089
Y3	21489	20850	162801	84489692	506	324	518	21.77	164903
Y4	21489	19709	197013	106671663	536	328	541	29.53	210689
Y5	21489	20696	213131	115425475	535	327	541	30.65	225931
Y6	21489	19586	94951	46924882	476	321	494	20.47	91667

Table S3. List of primers used in the present study

Items	Primer sequence (5'-3')	Annealing temp. (°C)	Reference
Total bacteria	F: ACTCCTACGGGAGGCAGCAG<br>R: ATTACCGCGGCTGCTGG	60	[1]
Firmicutes	F: GGAGYATGTGGTTTAATTCGAAGCA<br>R: AGCTGACGACAACCATGCAC	60	[1]
Bacteroidetes	F: GGARCATGTGGTTTAATTCGATGAT<br>R: AGCTGACGACAACCATGCAG	60	[1]
Bacteroides	F: GAGAGGAAGGTCCCCCAC<br>R: CGCTACTTGGCTGGTTCAG	60	[1]
Prevotella	F: CACRGTAAACGATGGATGCC<br>R: GGTCGGGTTGCAGACC	60	[2]
Blautia	F: GTGAAGGAAGAAGTATCTCGG<br>R: TTGGTAAGGTTCTTCGCGTT	55	[3]
Methanobrevibacter	F: TGGGAAACTGGGGATAATACTG<br>R: AATGAAAAGCCATCCCGTTAAG	60	[4]
Desulfovibrio	F: CCGTAGATATCTGGAGGAACATCAG<br>R: ACATCTAGCATCCATCGTTTACAGC	60	[5]
AcsB	F: CTBTGYGGDGCIGTIWSMTGG<br>R: AARCAWCCRCADGADGTCATIGG	52	[6]
McrA	F: TTCGGTGGATCDCARAGRGC<br>R: GBARGTCGWAWCCGTAGAATCC	60	[7]
AprA	F: TGGCAGATCATGATYMAYGG<br>R: GCGCCAACYGGRCCRTA	60	[8]

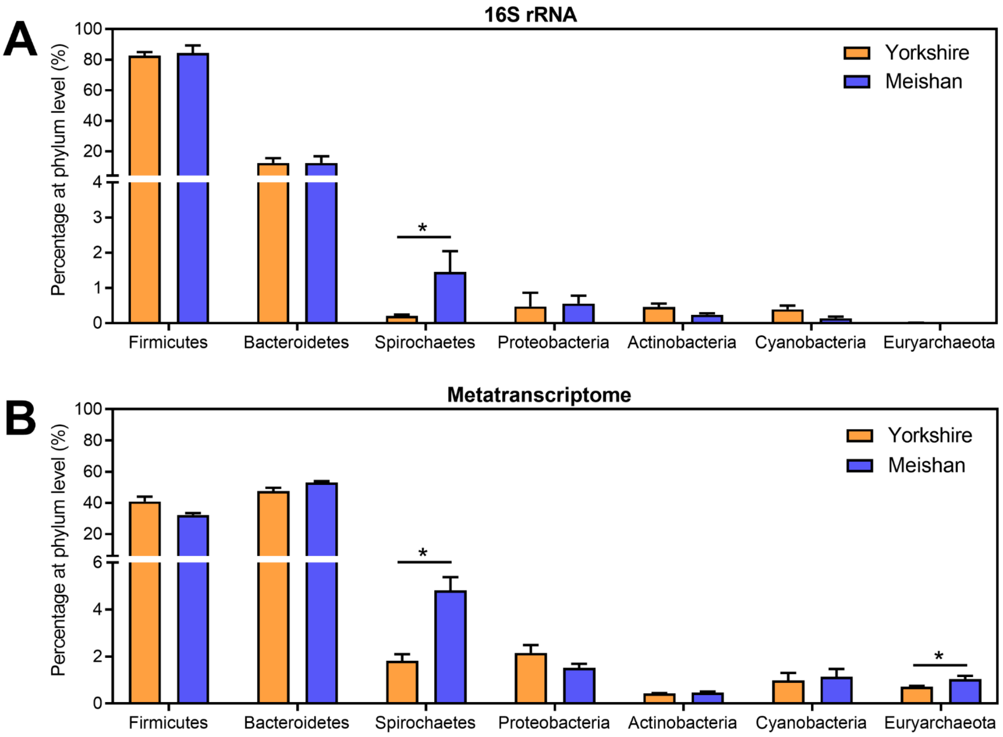

Figure S1. The relative abundances of colonic microbial phyla between Yorkshire and Meishan pigs based on 16S rRNA gene sequencing **(A)** and metatranscriptomics **(B)**. Data are expressed as means ± SEM. The Wilcoxon rank-sum test was used to analyze differences between Yorkshire and Meishan groups (n = 6 per group); *, adjusted $P < 0.05$.

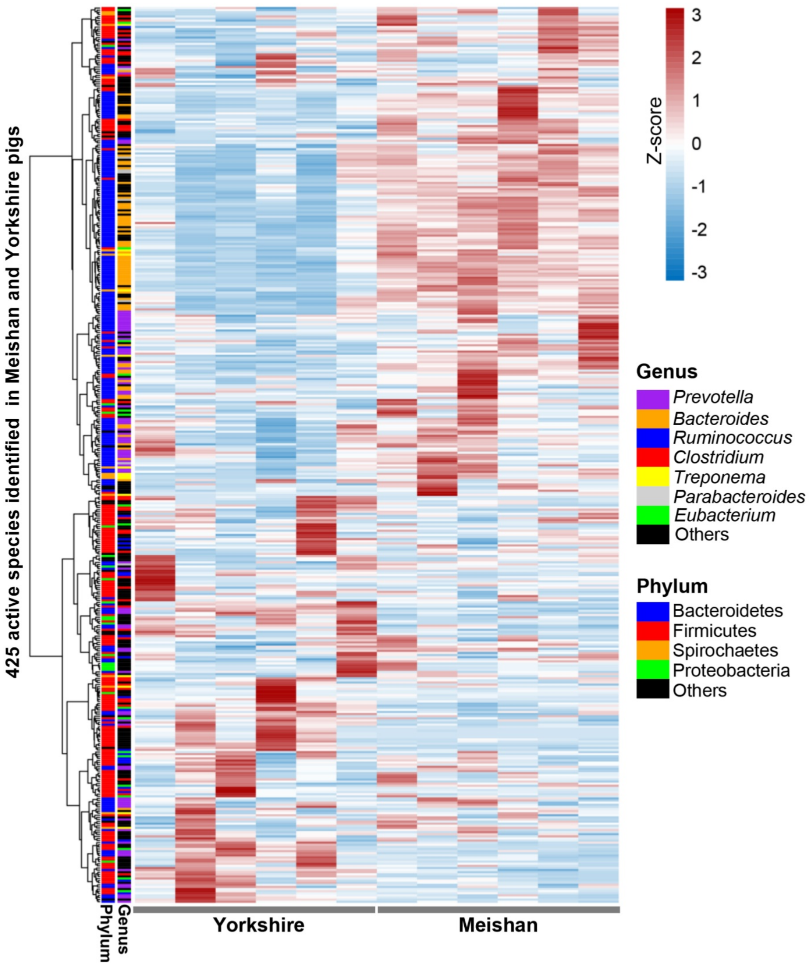

Figure S2. Heat map shows the 425 active microbial species (average relative abundance in at least one group of > 0.01% and being present in more than 50% of the samples in one group based on metatranscriptomic data) standardized using the Z-score method. Different-colored squares representing the active species were classified into different phyla and genera.

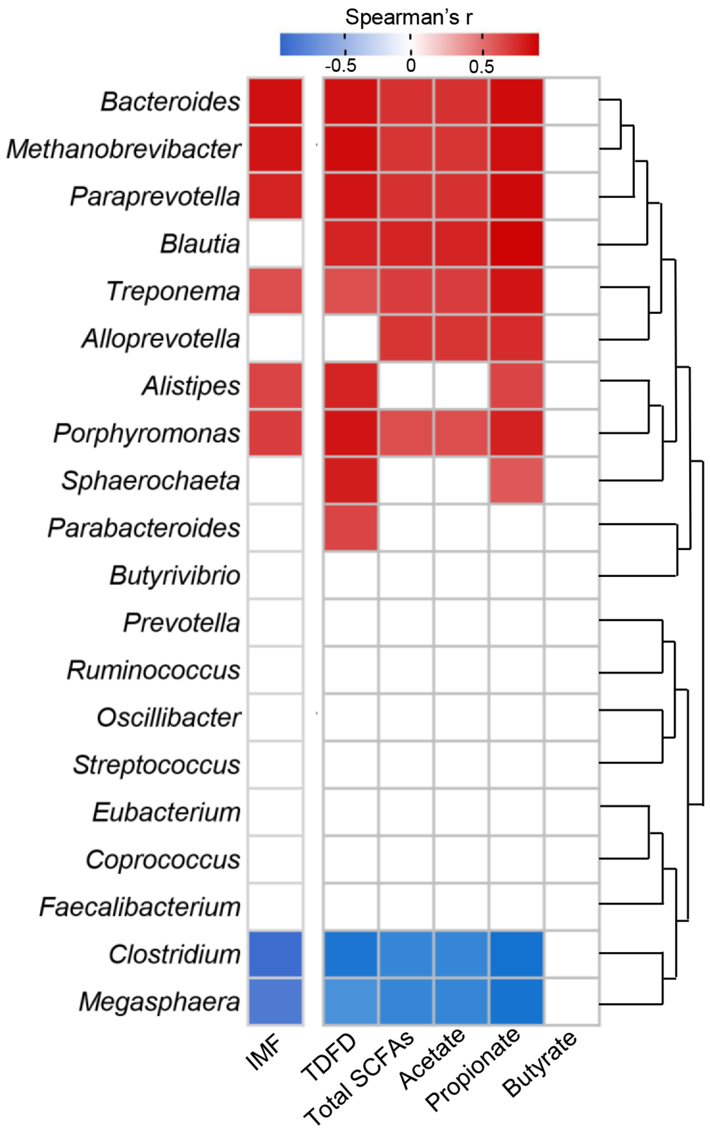

Figure S3. The relationships of fat deposition, fiber digestibility and SCFAs with microbial community at genus level (average relative abundance in at least one group of > 0.5 % based on metatranscriptomic data). Only Spearman's significance levels *P* < 0.05 are shown, red and blue colors represent positive or negative correction, respectively. The microbial genera were also clustered with a Spearman correlation coefficient. IMF, intramuscular fat; TDFD, digestibility of total dietary fiber.

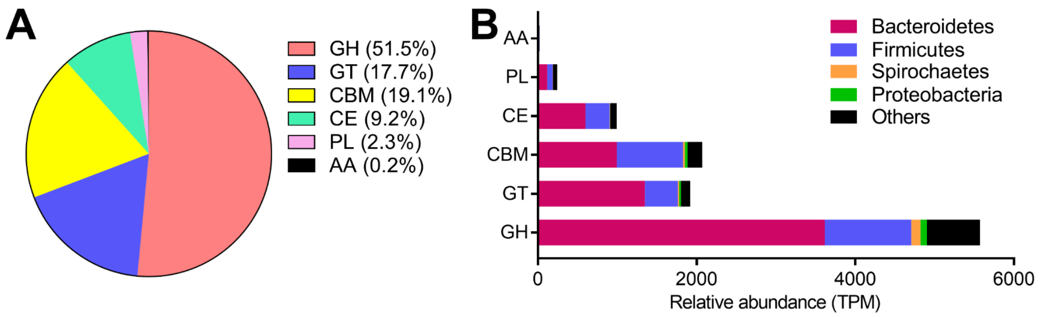

Figure S4. Distribution of different CAZyme classifications in the colonic microbiome (phylogenetic distribution of CAZyme familes assigned to the identified phyla).

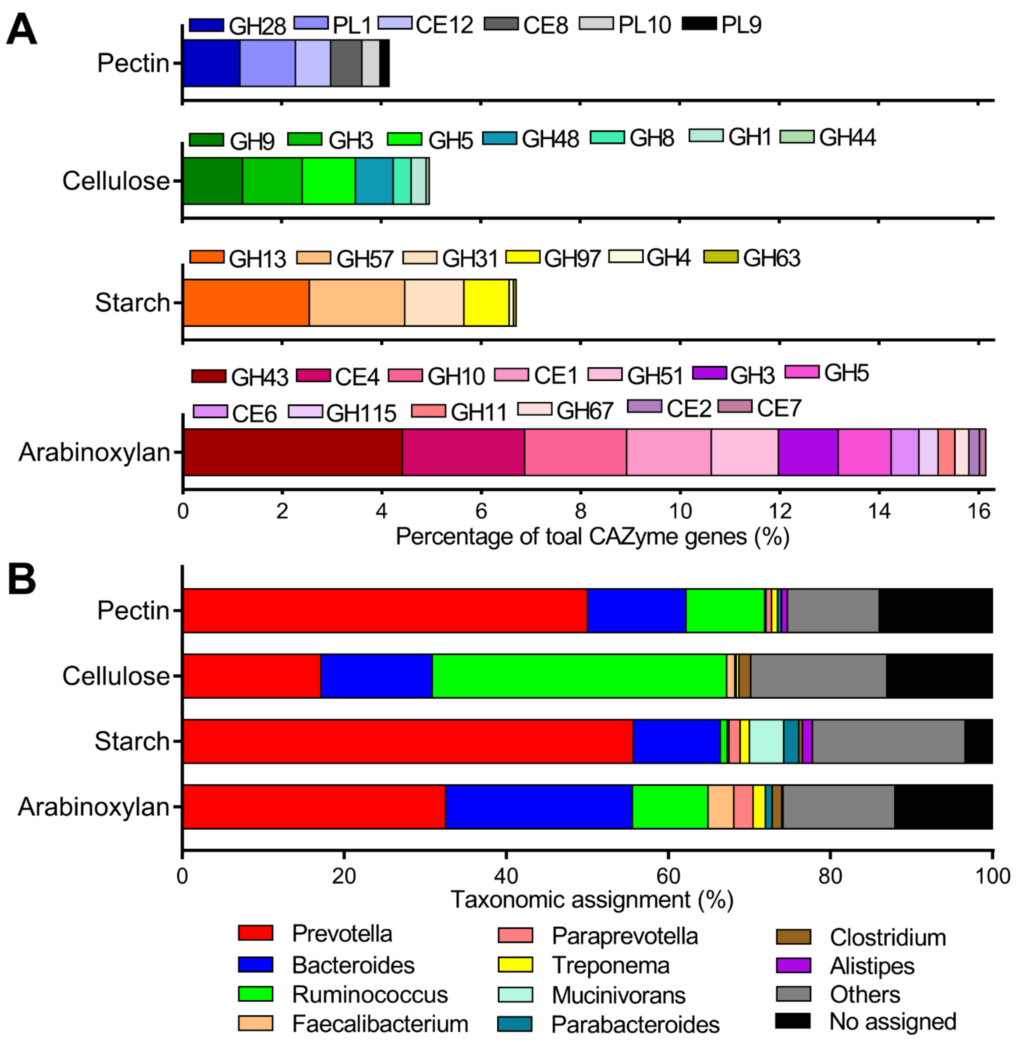

Figure S5. CAZyme gene expressions in the colonic microbiome of pigs. **(A)** The distribution and percentage of CAZyme families related to the utilization of arabinoxylan, starch, cellulose and pectin. **(B)** Taxonomic classification of transcripts involved in the degradation of different carbohydrate substrates. The percentage is defined as the proportion of transcripts from given subgenus in the total of CAZyme (GH, CE and PL only) transcripts targeting individual polysaccharide component as indicated. No assigned means there is no specific taxonomic information at the genus level.

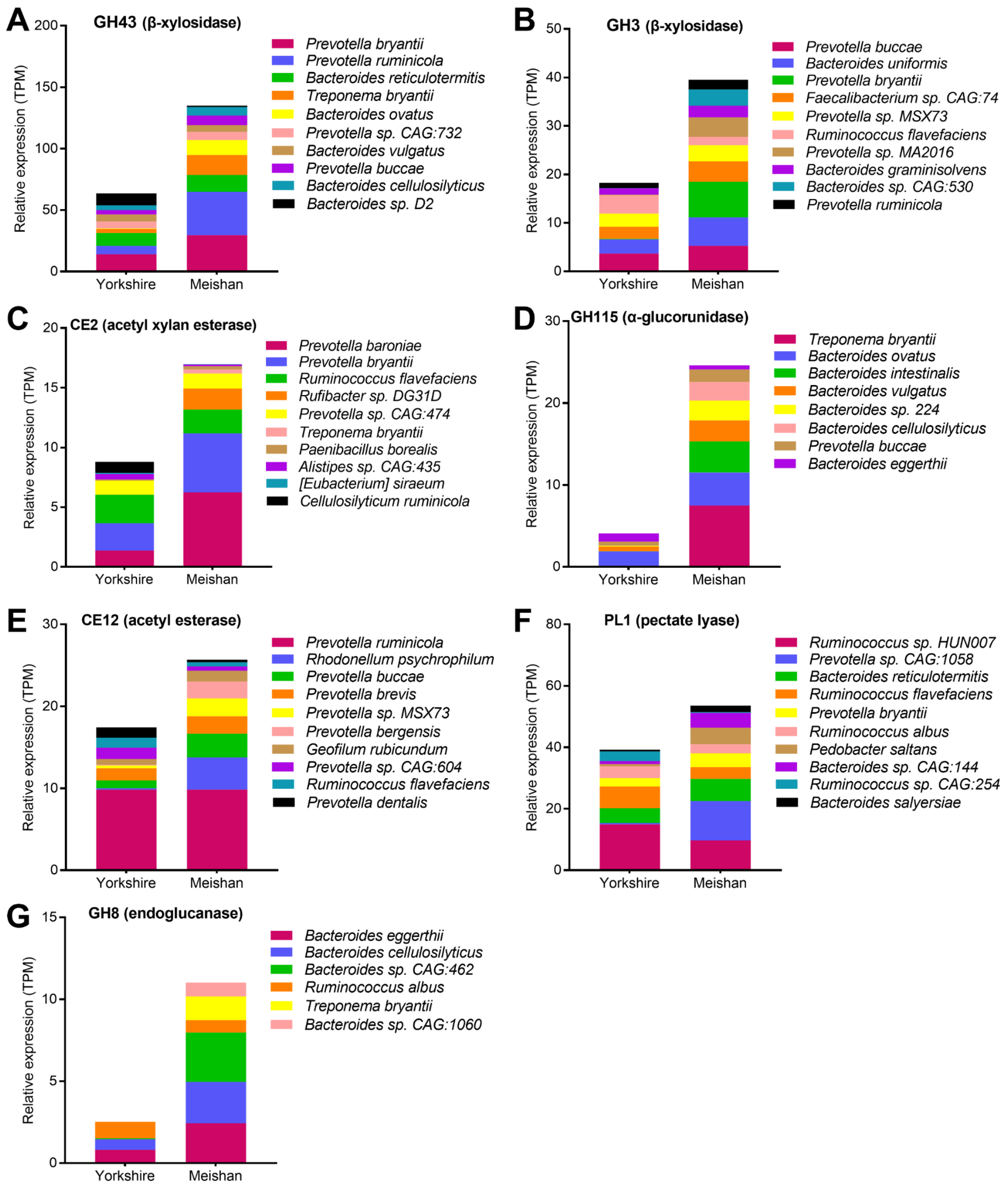

71
72 **Figure S6.** Phylogenetic distribution of differentially expressed CAZymes assigned to
73 the identified species in colonic microbiome between Yorkshire pigs and Meishan pigs.
74

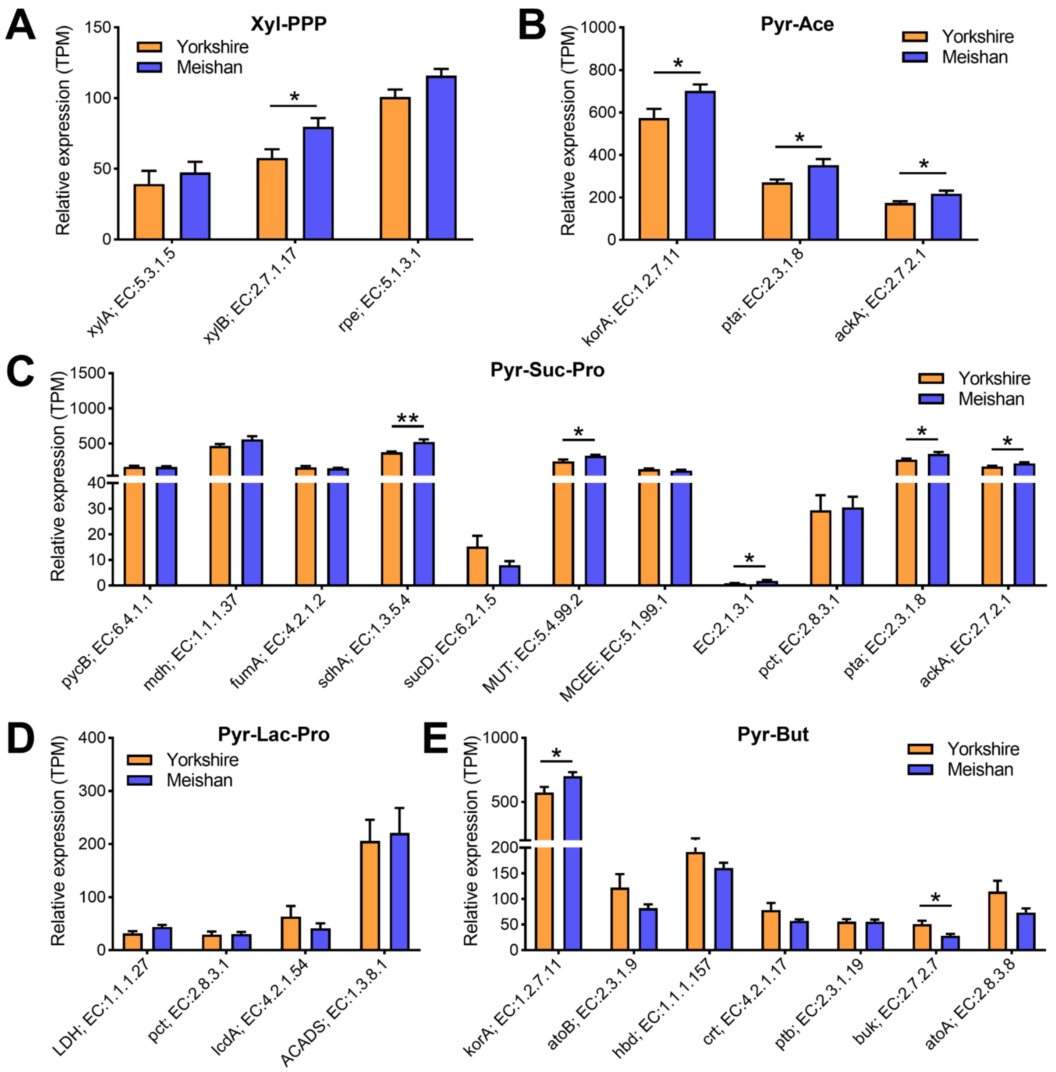

Figure S7. The relative expression levels of enzymes involved in xylose utilization and SCFA production between Meishan and Yorkshire pigs. **(A)** Enzymes in catabolic pathway of xylose into pentose phosphate pathway (Xyl-PPP). **(B)** Enzymes in acetate synthesis from pyruvate (Pyr-Ace). **(C)** Enzymes in pyruvate fermentation to propionate via succinate (Pyr-Suc-Pro). **(D)** Enzymes in pyruvate fermentation to propionate via lactate (Pyr-Lac-Pro). **(E)** Enzymes in butyrate synthesis from pyruvate (Pyr-But). Data are expressed as means ± SEM. The Wilcoxon rank-sum test was used to analyze differences between Yorkshire and Meishan groups (n = 6 per group). *$P < 0.05$, **$P < 0.01$.

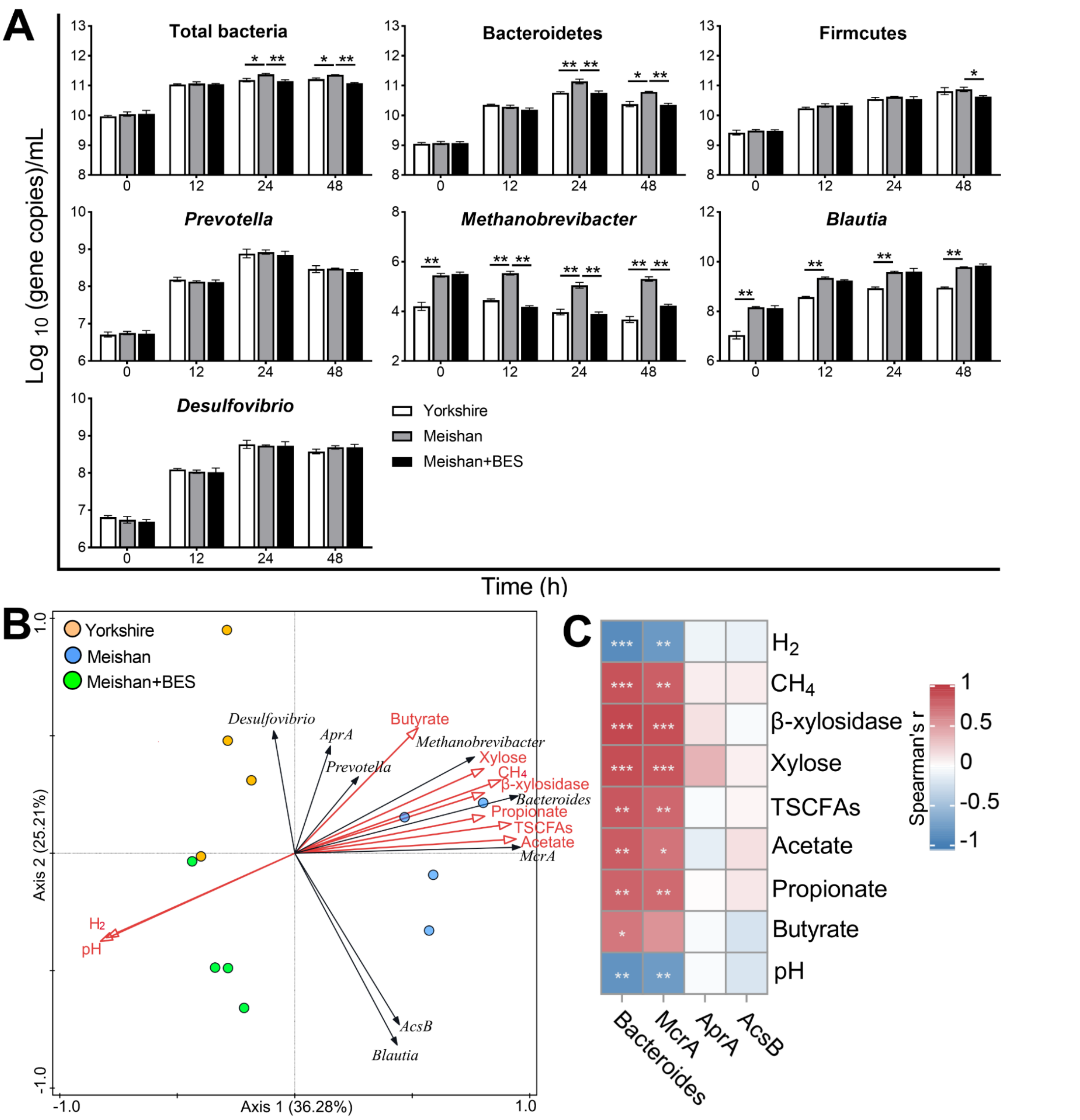

Figure S8. Quantification of representative microbes associated with arabinoxylan degradation and H_2 uptake during *in vitro* fermentation. **(A)** qPCR measurement of total bacterial 16S rRNA genes, representative microbial phyla and genera during fermentation among three groups (n = 4 per group). **(B)** RDA plots of major microbial community and environmental factors at 24 h. Red arrows indicate the environmental factors, and black arrows indicate the microbial genera and hydrogenotrophic functional groups. **(C)** Correlation analyses of copy numbers of *Bacteroides* and hydrogenotrophic functional groups with fermentation parameters at 24 h of inoculation. Data are expressed as means ± SEM. An unpaired two-tailed Student's t-test and the Spearman's method were used to assess differences; *$P < 0.05$, **$P < 0.01$, ***$P < 0.001$.

Supplementary References

1. Guo X, Xia X, Tang R, Zhou J, Zhao H, Wang K. Development of a real-time PCR method for Firmicutes and Bacteroidetes in faeces and its application to quantify intestinal population of obese and lean pigs. Lett Appl Microbiol. 2008;**47**:367-73. https://doi.org/10.1111/j.1472-765X.2008.02408.x.
2. Harmsen HJ, Raangs GC, He T, Degener JE, Welling GW. Extensive set of 16S rRNA-based probes for detection of bacteria in human feces. Appl Environ Microbiol. 2002;**68**:2982-90. https://doi.org/10.1128/aem.68.6.2982-2990.2002.
3. Kurakawa T, Ogata K, Matsuda K, Tsuji H, Kubota H, Takada T et al. Correction: diversity of intestinal Clostridium coccoides group in the Japanese population, as demonstrated by reverse transcription-quantitative PCR. PLoS One. 2016;**11**:e0152753. https://doi.org/10.1371/journal.pone.0152753.
4. Ufnar JA, Wang SY, Christiansen JM, Yampara-Iquise H, Carson CA, Ellender RD. Detection of the nifH gene of Methanobrevibacter smithii: a potential tool to identify sewage pollution in recreational waters. J Appl Microbiol. 2006;**101**:44-52. https://doi.org/10.1111/j.1365-2672.2006.02989.x.
5. Fite A, Macfarlane GT, Cummings JH, Hopkins MJ, Kong SC, Furrie E et al. Identification and quantitation of mucosal and faecal desulfovibrios using real time polymerase chain reaction. Gut. 2004;**53**:523-9. https://doi.org/10.1136/gut.2003.031245.
6. Gagen EJ, Denman SE, Padmanabha J, Zadbuke S, Al Jassim R, Morrison M et al. Functional gene analysis suggests different acetogen populations in the bovine rumen and tammar wallaby forestomach. Appl Environ Microbiol. 2010;**76**:7785-95. https://doi.org/10.1128/aem.01679-10.

7. Denman SE, Tomkins NW, McSweeney CS. Quantitation and diversity analysis of ruminal methanogenic populations in response to the antimethanogenic compound bromochloromethane. FEMS Microbiol Ecol. 2007;**62**:313-22. https://doi.org/10.1111/j.1574-6941.2007.00394.x.

8. Meyer B, Kuever J. Molecular analysis of the diversity of sulfate-reducing and sulfur-oxidizing prokaryotes in the environment, using aprA as functional marker gene. Appl Environ Microbiol. 2007;**73**:7664-79. https://doi.org/10.1128/aem.01272-07.
